# Supplementary material for: Covariant spatio-temporal receptive fields for spiking neural networks
Source: Nat Commun. 2025 Sep 5;16:8231. doi: 10.1038/s41467-025-63493-0 (PMC12413456; doi:10.1038/s41467-025-63493-0)
Supplement: Supplementary file 1 — Supplementary Information [file 41467_2025_63493_MOESM1_ESM.pdf]

Supplementary material for "Covariant spatio-temporal receptive fields for spiking neural networks"

J. E. Pedersen, J. Conradt, T. Lindeberg
Computational Science and Technology
KTH Royal Institute of Technology
Stockholm
{jeped@kth.se}

A Covariance properties in space and time

Definition A.1 (Covariance and invariance)**.** Given a set $\mathcal{X}$, the map $\phi : \mathcal{X} \to \mathcal{X}$ is *covariant* if it obeys $\phi(g \cdot x) = g' \cdot \phi(x)$ for all actions g, g' over $\mathcal{X}$. That is, there is a sense in which the map ϕ applied *before* the group action is comparable to its application *after* the group action. This is also known as *equivariance.* If g acts *trivially* on $\mathcal{X}$, then $\phi(g \cdot x) = \phi(\mathbb{1} \cdot x) = \phi(x) = \mathbb{1}(\phi \cdot x) = g(\phi \cdot x)$ and we call ϕ *invariant* to g.

Definition A.2 (Translation)**.** A shift operation ($\phi_{translate}$) translates a signal x by a translation offset Δx according to $(\Delta x, x) = x - \Delta x$.

Definition A.3 (Scaling)**.** The scaling operation (ϕ_{scale}) scales a signal x by a spatial scaling factor s defined by $(s, x) = sx$.

Definition A.4 (Rotation)**.** Rotations rotate a signal x around its origin by an angle θ. In two dimensions, when $x \in \mathbb{R}^2$, we describe this in matrix form

$$(\theta, x) = \left(\theta, \begin{bmatrix} x_1 \\ x_2 \end{bmatrix}\right) = \begin{bmatrix} \cos\theta & -\sin\theta \\ \sin\theta & \cos\theta \end{bmatrix} \begin{bmatrix} x_1 \\ x_2 \end{bmatrix} \tag{1}$$

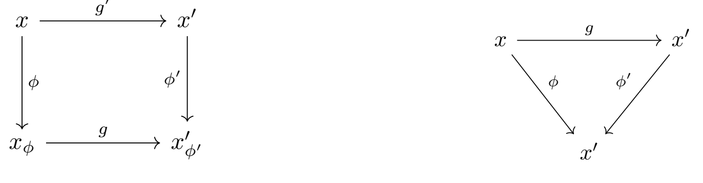

(a) If $\phi : \mathcal{X} \to \mathcal{X}$ is covariant to the actions g and g' acting on some signal domain $\mathcal{X}$, then $g \cdot \phi = \phi' \cdot g'$, given some $x, x', x_\phi, x'_\phi \in \mathcal{X}$.

(b) If $\phi : \mathcal{X} \to \mathcal{X}$ is invariant to the action g on some signal $\mathcal{X}$, then $\phi' \cdot g = \phi$, given some $x, x', x'' \in \mathcal{X}$.

Supplementary Figure 1: Covariance (left) retains information about the actions g, g' after their application in some form, while invariance (right) discards it.

Definition A.5 (Shearing)**.** The shear operation (ϕ_{shear}) translates a signal x parallel to a given line. In two dimensions, the shear operation (λ, x) can be defined using the shear matrix parallel to either the horizontal

$$(\lambda_x, x) = \left((\lambda_x, 0), \begin{bmatrix} x_1 \\ x_2 \end{bmatrix}\right) = \begin{bmatrix} 1 & \lambda_x \\ 0 & 1 \end{bmatrix} \begin{bmatrix} x_1 \\ x_2 \end{bmatrix} \tag{2}$$

or vertical

$$(\lambda_y, x) = \left((0, \lambda_y), \begin{bmatrix} x_1 \\ x_2 \end{bmatrix}\right) = \begin{bmatrix} 1 & 0 \\ \lambda_y & 1 \end{bmatrix} \begin{bmatrix} x_1 \\ x_2 \end{bmatrix} \tag{3}$$

axis.

Definition A.6 (Affine transformation)**.** We define an affine transformation as any map $\psi : \mathcal{V} \mapsto \mathcal{V}$ that sends a point in a vector space $\mathcal{V}$ to another point in that same vector space. Affine transformations are the most general type of linear transformation, and include arbitrary compositions of translations, scalings, rotations, and shearings. For image transformations in two dimensions, an affine transformation in matrix form ($\mathcal{A}$) is given by

$$(\mathcal{A}, x) = \left(\mathcal{A}, \begin{bmatrix} x_1 \\ x_2 \end{bmatrix}\right) = \begin{bmatrix} a_{11} & a_{12} \\ a_{21} & a_{22} \end{bmatrix} \begin{bmatrix} x_1 \\ x_2 \end{bmatrix} \tag{4}$$

Definition A.7 (Galilean transformation)**.** Newtonian physics tells us that two reference frames with an N-dimensional spatial component ($x \in \mathbb{R}^N$) and temporal component ($t \in \mathbb{R}$) are related according to

$$\begin{aligned} x' &= x + vt \\ t' &= t \end{aligned} \tag{5}$$

where v is the relative velocity for each dimension $n \in N$. In the case of two spatial dimensions ($n = 2$) and one temporal dimension, we describe the relationship between two points p in space-time as follows [1, Eq. (20)]

$$(v, p') = \begin{bmatrix} x_1' \\ x_2' \\ t' \end{bmatrix} = \begin{bmatrix} 1 & 0 & v_1 \\ 0 & 1 & v_2 \\ 0 & 0 & 1 \end{bmatrix} \begin{bmatrix} x_1 \\ x_2 \\ t \end{bmatrix} = G\ p \tag{6}$$

Following the above definitions of covariance and transformations, we distinguish between invariance as well as covariance properties for affine *spatial* transformations, *temporal* scaling, and *spatio-temporal* Galilean transformations.

B Covariance properties in scale-space representations

Scale-space theory provides a means to represent signals at varying spatial or temporal scales. The covariance properties of the scale-space representations arrive from the commutative relationships shown in Figure 2, which we will elaborate and expand below. Given a continuous signal $f(t)\colon \mathbb{R} \to \mathbb{R}$, we define the scale-dependent scale-space representation L, parameterized by a scale $\tau \in \mathbb{R}_+\colon L_g(t, \tau)$, as the convolution integral with scale-dependent kernels $g\colon \mathbb{R} \times \mathbb{R}_+ \to \mathbb{R}$ [2]

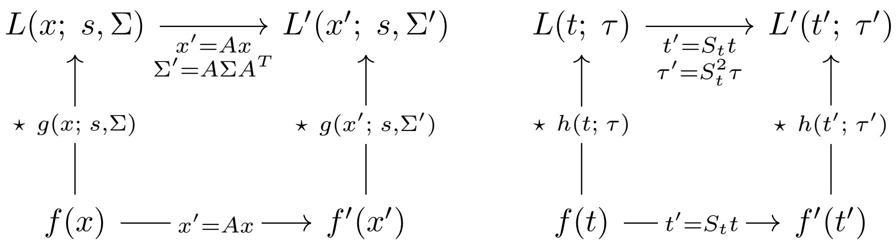

Supplementary Figure 2: The relation of a signal (f) and its scale-space representation (L) under spatial affine transformations (left) and temporal scaling transformations (right).

$$L_g(t,\tau) = g(t) \star f(t) = (g \star f)(t) = \int_{-\infty}^{\infty} g(\xi;\tau)\ f(t-\xi)\ d\xi \tag{7}$$

For this property to hold, the kernel g must *retain or diminish* variations, such that the changes in sign values for any signal sequence following the kernel application is less than or equal to the number of changes in sign value before that kernel has been applied. Specifically, if we, following Schoenberg [3], define a "sign change detection function" $V\colon (\mathbb{R} \to \mathbb{R}) \to \mathbb{N}$, that finds the number of times a function f changes sign given the sequence $\{f(t_0), f(t_1), ..., f(t_n)\}$, and require that

$$V(g \star f) \leqslant V(f) \tag{8}$$

This condition is met if and only if g has a bilateral Laplace transform of the form

$$\int_{-\infty}^{\infty} e^{-st} g(t) dt = C\, e^{\gamma s^2 + \delta s} \prod_{i=1}^{\infty} \frac{e^{a_i s}}{1 + a_i s} \tag{9}$$

for $-c < Re(s) < c$, $c > 0$, $\gamma \geqslant 0$, $C \neq 0$, δ, a_i are real, and assuming $\sum_{i=1}^{\infty} a_i^2$ converges [3]. Over the spatial domain, the Gaussian kernel for some signal x and covariance matrix Σ

$$g(x;\Sigma) = \frac{1}{2\pi\ \sqrt{\det\Sigma}} e^{-x^T\Sigma^{-1}x/2} \tag{10}$$

along with its derivatives, is known to meet the above conditions [4]. Concerning the temporal domain, it was shown by Lindeberg and Fagerström [5] that if we require the temporal kernels to be time-causal, in the sense that they do not access values from the future in relation to any time moment, then convolutions with truncated exponential kernels [2, Eq. (8)]

$$h_{composed}(\cdot;\ \mu) = *_{k=1}^{K} h_{exp}(\cdot;\ \mu_k) \tag{11}$$

by both necessity and sufficiency constitute the class of time-causal temporal scale-space kernels.

B.1 Temporal scale covariance for the causal limit kernel

The Gaussian kernel does not immediately apply to the temporal domain because it relies on future, unseen, signals, which is impossible in practice [6]. Instead, based on the above cited classification of time-causal scale-space kernels [5, 7, 2], convolutions with truncated exponential kernels h_{exp}

$$h_{exp}(t, \mu_k) = \begin{cases} \frac{1}{\mu_k} e^{-t/\mu_k} & t > 0 \\ 0 & t \leqslant 0 \end{cases} \tag{12}$$

coupled in cascade, where μ_k are temporal time constants, lead to the formulation of a temporal scale-space representation.

Note that we assume normalization, such that $\int_{-\infty}^{\infty} h(t;\ \tau)\, dt = 1$. By composing K truncated exponential kernels in cascade, in the limit when K trends to ∞, Lindeberg [7, Eq. (38)] introduces a "limit kernel" Ψ in the Fourier domain

$$\begin{aligned} \hat{\Psi}(\omega; \tau, c) &= \lim_{K \to \infty} \hat{h}(\omega; \tau, c, K) \\ &= \prod_{k=1}^{\infty} \frac{1}{1 + ic^{-k}\sqrt{c^2 - 1}\sqrt{\tau}\omega} \end{aligned} \tag{13}$$

where the temporal scaling parameter, τ_k for $k \in K$ scale levels, is distributed according to integer powers of the distribution parameter c following $\tau_k = c^{2(k-K)}\, \tau$ for $c > 1$. Scaling $\hat{\Psi}$ with some factor S_t (t denoting time), we see that [7, Eq. (43)]

$$\begin{aligned} \hat{\Psi}(\frac{\omega}{S_t}; S_t^2 \tau, c) &= \prod_{k=1}^{\infty} \frac{1}{1 + ic^{-k}\sqrt{c^2 - 1}\sqrt{S_t^2 \tau}\frac{\omega}{S_t}} \\ &= \hat{\Psi}(\omega; \tau, c) \end{aligned} \tag{14}$$

The scale-space representation of a given signal f by a scaling factor $S_t = 1/c$ such that $t' = t/c$ and $\tau' = \tau/c^2$ is then [7, Eq. (45)]

$$L'(t';\ \tau', c) = \left(\Psi\left(\cdot;\ \frac{\tau}{c^2},\ c\right) \star f'(\cdot)\right)(t'; \tau' -,\ c) \tag{15}$$

Covariance between smoothing representations L and L' follows, again under logarithmic distribution of scales

$$L(t; \tau, c) = L'(t'; \tau', c) \quad \text{for } t' = c^j t \text{ and } \tau' = c^{2j}\tau \tag{16}$$

where τ is the *temporal* scale and S_t denotes a temporal rescaling factor $S_t = c^j$, $j \in \mathbb{Z}$. Returning to the temporal domain, this implies a direct relationship between neighboring temporal scales [1, Eq. (41)]

$$\Psi(t; \tau, c) = h\left(t; \frac{\sqrt{c^2 - 1}}{c}\sqrt{\tau}\right) \star \Psi(\cdot; \frac{\tau}{c^2}, c) \tag{17}$$

with the following scaling property

$$S\Psi(S_t\, t; S_t^2 \tau, c) = \Psi(t; \tau', c) \tag{18}$$

Ψ can be understood as a *scale-covariant time-causal limit kernel*, because it "obeys a closedness property over all temporal scaling transformations $t' = S^2 t$ with temporal rescaling factors $S = c^j$ $(j \in \mathbb{Z})$ that are integer powers of the distribution parameter c" [1, eq. 47].

B.2 Covariance over joint spatial and temporal transformations

We now want to derive a single kernel that is provably covariant to spatial affine and Galilean transformations as well as temporal scaling transformations. With the addition of time, the spatial and temporal signals are no longer separable. Instead, we look to Newtonian physics, where relative motions between two frames of reference are described by Galilean transformations according to (5). Directly building on the (spatial) Gaussian kernel (10) and the time-causal limit kernel (17), we can, following [7], establish spatio-temporal receptive fields as their composition

$$T(x, t; \Sigma, v, \tau, c) = g(x; \Sigma)\ \Psi(t; \tau, c) \tag{19}$$

The corresponding scale-space representation is

$$L(x, t; \Sigma, v, \tau, c) = \iint_{\xi \in \mathbb{R}^2} \int_{\rho \in \mathbb{R}} T(\xi, \rho; \Sigma, v, \tau, c)\ f(x - \xi, t - \rho)\ d\xi\ d\rho \tag{20}$$

Consider two spatio-temporal signals (video sequences) f, f' that are related according to a spatial affine transformation, a Galilean spatio-temporal transformation, and a temporal scaling transformation as follows

$$x' = A(x + wt) \quad \text{and} \tag{21}$$

$$t' = S_t\ t \tag{22}$$

According to Equation 21 in the main text

$$\Sigma' = A\, \Sigma\, A^T \tag{23}$$

we have

$$\begin{aligned} \Sigma'^{-1} &= (A \Sigma A^T)^{-1} = A^{-T} \Sigma^{-1} A^{-1} \quad \text{and} \\ \det \Sigma' &= \det\big(A \Sigma A^T\big) = |\det A|^2 \det \Sigma \end{aligned} \tag{24}$$

If we further set the relationships of two signals subject to Galilean and affine transformations as follows

$$\begin{aligned} v &= -A^{-1} w + A^{-1} v' S_t \\ Av &= -w + v' S_t \\ v' &= (Av + w)/S_t \end{aligned} \tag{25}$$

we can rewrite the Gaussian kernel (10) under Galilean and affine transformations as

$$
\begin{aligned}
g(x'-v't';\Sigma') &= \frac{1}{2\pi\sqrt{\det\Sigma'}}\exp\big\{\big(-(x'-v't')^T\Sigma'^{-1}(x'-v't')/2\big)\big\} \\
&= \frac{1}{2\pi|\det A|\sqrt{\det\Sigma}}\exp\big(-(A(x+wt)-((Av+w)/S_t)S_t t)^T A^{-T} \\
&\qquad \Sigma^{-1}A^{-1}(A(x+wt)-((Av+w)/S_t)S_t)/2\big) \\
&= \frac{1}{2\pi|\det A|\sqrt{\det\Sigma}}\exp\big(-(x-vt)^T\Sigma^{-1}(x-vt)/2\big)
\end{aligned} \tag{26}
$$

For f' we have the corresponding spatio-temporal scale-space representation as in (20)

$$
L'(x',t';\Sigma',v',\tau',v') = \int\int_{\xi'\in\mathbb{R}^2}\int_{\rho'\in\mathbb{R}} T'(\xi',\rho';\Sigma',v',\tau',c')f'(x'-\xi',t'-\rho')d\xi' d\rho' \tag{27}
$$

We now exchange variables according to (21) and (22) to find

$$
\begin{aligned}
d\xi' &= |\det A| d\xi \\
d\rho' &= S_t d\rho
\end{aligned} \tag{28}
$$

and, therefore,

$$
\begin{aligned}
L'(x',t';\Sigma',v',\tau',v') &= \int\int_{\xi'\in\mathbb{R}^2}\int_{\rho'\in\mathbb{R}} T'(\xi',\rho';\Sigma',v',\tau',c')f'(x'-\xi',t'-\rho')d\xi' d\rho' \\
&= \int\int_{\xi\in\mathbb{R}^2}\int_{\rho\in\mathbb{R}} T(\xi,\rho;\Sigma,v,\tau,c)f(x-\xi,t-\rho)d\xi d\rho \\
&= L(x,t;\Sigma,v,\tau,v)
\end{aligned} \tag{29}
$$

which establishes the desired covariance property for the spatio-temporal scale-space representation L under affine, Galilean, and temporal scaling transformations, as shown in Figure 1 in the main text. This result also holds for the temporal Gaussian kernel (10) or truncated exponential kernel (12) by relating $\tau' = S_t^2\tau$.

B.3 Temporal scale covariance for parallel first-order integrators

Consider a parallel set of temporal K scale channels with a single time constant for each parallel timescale "channel", modelled as a truncated exponential kernel as in equation (12):

$$
h_{composed}(\cdot;\tau_k) = h_{exp}(\cdot;\mu_k) \tag{30}
$$

for $\mu_k = \sqrt{\tau_k}$ [8, Eq. (16)] Each "channel" would assign a unique temporal scale-representation of the initial signal with a delay and duration, parameterized by

μ_k. We denote a temporal scale-space channel representation T for some signal $f\colon \mathcal{X} \to \mathcal{X}$

$$T(t;\mu) = \int_{u=0}^{\infty} f(t-u)\, h_{exp}(u;\mu)\; du \tag{31}$$

and observe the direct relation under a temporal scaling operation $t' = St$ for two functions $f'(t') = f(t)$

$$\begin{aligned} T(t';\mu') &= \int_{u'=0}^{\infty} f'(t'-u')\frac{1}{\mu'}e^{-t'/\mu'}du' \\ &\overset{1}{=} \int_{u=0}^{\infty} f'\left(S(t-u)\right)\frac{1}{S\mu}e^{-St/S\mu}Sdu \\ &= \int_{u=0}^{\infty} f(t-u)\frac{1}{\mu}e^{-t/\mu}du \\ &= T(t;\mu) \end{aligned} \tag{32}$$

where step (1) sets $u' = Su$, $du' = Sdu$, and $\mu' = S\mu$. A single filter T is thus scale-covariant for the scaling operation S, and a *set* of filters ($h_{composed}$) will be scale-covariant over multiple scales, provided the logarithmic distribution of K scales μ_k as in equation (16), and for scaling factors S that are integer powers of the ratio between the time constants μ_k of adjacent scale levels.

C Temporal scale covariance for leaky integrators

This section establishes temporal scale covariance for leaky integrators by demonstrating that they are special cases of a first-order integrator, after which the temporal scale covariance guarantees for first-order integrators apply, as in Section B.3.

A linear leaky integrator describes the evolution of a voltage u characterized by the following first-order integrator

$$\mu\frac{du}{dt} = -(u-u_0) + RI \tag{33}$$

where μ is a time constant controlling the speed of the integration, u_0 is the "resting state", or target value of the neuron it will leak towards, R is a linear resistor, and I describes the driving current [9]. Assuming $u_0 = 0$ and $R = 1$ we arrive at [9, 10]

$$u(t;\mu) = \frac{1}{\mu}\int_{0}^{\infty} e^{-\xi/\mu}\, I(t-\xi)\; d\xi \tag{34}$$

This is precisely the first-order integrator in the form of equation (12), and the temporal scale covariance from equation (32) follows.

D Temporal scale covariance for thresholded leaky integrators

This section establishes temporal scale covariance for thresholded linear leaky integrators. We first establish the usual, discrete formulation of thresholded leaky integrator-and-fire models and later introduce the spike response model that generalizes the neuron model as a set of connected filters. Finally, we build on the proof for the causal first-order leaky integrator above to establish temporal scale covariance for leaky integrate-and-fire models.

The leaky integrate-and-fire model is essentially a leaky integrator with an added threshold [9], which discretizes the activation using the Heaviside function H

$$H(x; \theta_{thr}) = \begin{cases} 1, & x \geqslant \theta_{thr} \\ 0, & x < \theta_{thr} \end{cases} \tag{35}$$

parameterized over some threshold value θ_{thr}. When $H = 1$, we say that the neuron "fires", whereupon the integrated voltage (its membrane potential) resets to θ_{reset}:

$$u(t; \mu) = \theta_{reset} \tag{36}$$

The total dynamics of the leaky integrate-and-fire neuron model can be summarized as three equations capturing the subthreshold dynamic from equation (34), the thresholding from equation (35), and the reset from equation (36) [9].

It is worth noting that the threshold in the continuous case is a simplification of the dirac delta function defined as

$$\delta(t) = \lim_{\sigma \to 0} g(t; \sigma) \tag{37}$$

where g is a normalized Gaussian kernel. Integrating over time, we therefore have

$$\int_{-\infty}^{\infty} \delta(t)\ dt = 1 \tag{38}$$

Hence the "spike" ($H = 1$) in equation (35): any layer following a thresholded activation function will be subject to a series of these unit impulses over time. As such, the neuron activation output function can be written as a sum of N unit impulses over time [9]

$$\Gamma(t) = \sum_{n=1}^{N} \delta(t - t_n) \tag{39}$$

D.1 Spike response model

This formulation generalizes to numerous neuron models, but we will restrict ourselves to the LIF equations, which can be viewed as a composition of three filters: a membrane filter (κ), a threshold filter (H), and a membrane reset filter (η') shown in Figure 3. The spike response model defines neuron models as a composition of parametric functions (filters) of time [9]. The membrane filter and membrane reset filter describes the subthreshold dynamics as follows:

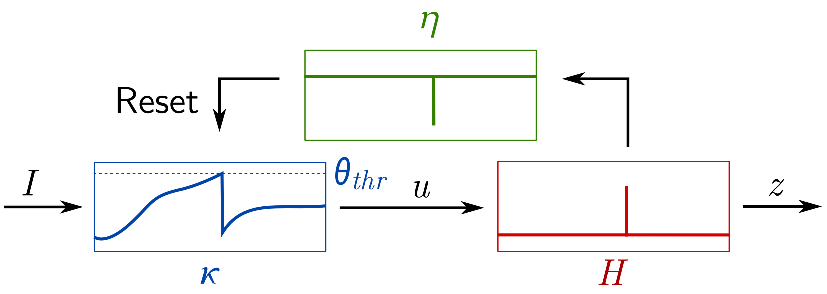

Supplementary Figure 3: The spike response model recasts the leaky integrate-and-fire model as a composition of three filters: a membrane filter in blue (κ), a threshold filter in red (H), and a reset filter in green (η). The graphs inside the filters depict their respective outputs over time using the kernels in (40), given some input signal, I.

$$u(t) = \int_0^\infty \eta'(s)\,\Gamma(t-s) + \kappa(s)\,I(t-s)\,ds \tag{40}$$

The reset filter, η', can be understood as the resetting mechanism of the neuron, that is, the function controlling the subthreshold behaviour immediately after a spike. This contrasts the LIF formalism from equation (36) because the resetting mechanism is now a function of time instead of a constant.

Since the resetting mechanism depends entirely on the time of the threshold activations, we can recast it to a function of time (t), where t_f denotes the time of the previous spike

$$u(t) = \eta(t - t_f) + \int_0^\infty \kappa(s) I(t-s) ds \tag{41}$$

Following [9, Eq. (6.33)] we define η more concretely as a linearized reset mechanism for the LIF model

$$\eta(t - t_f; \mu_r) = -\theta_{thr}\; e^{-(t-t_f)/\mu_r} \tag{42}$$

The above equation illustrates how the "after-spike effect" decays at a rate determined by μ_r. Initially, the kernel corresponds to the *negative* value of θ_{thr} at the time of the spike, effectively resetting the membrane potential by θ_{thr}. In the LIF model, the reset is instantaneous, which we observe when $\mu_r \to 0$. This is visualized in Figure 3 where the set of filters correspond to the subthreshold mechanism in equation (34) (which we know to be scale covariant from equations (32)), the Heaviside threshold in equation (35), and the reset mechanism in equation (42).

We arrive at the following expression for the subthreshold voltage dynamics of the LIF model:

$$z(t) = -\theta_{thr}\; e^{-(t-t_f)/\mu_r} + \int_0^\infty \kappa(s)\,I(t-s)\,ds \tag{43}$$

D.2 Temporal covariance for the LIF model

We are now ready to state the scale-space representation T of a LIF model as a special case of the above equation (43) where $\kappa \coloneqq h_{exp}$:

$$T(t; \mu, \mu_r) = -\theta_{thr}\ e^{-(t-t_f)/\mu_r} + \int_0^\infty f(t-z)\, h_{exp}(z; \mu)\, dz \tag{44}$$

Considering a temporal scaling operations $t' = St$ and $t'_f = St'_f$ for the functions $f'(t') = f(t)$, we follow the steps in equation (32):

$$\begin{aligned} T(t'; \mu', \mu'_r) &= -\theta_{thr}\ e^{-(t'-t'_f)/\mu'_r} + \int_{z'=0}^\infty f'(t'-z')\, \frac{1}{\mu'} e^{-t'/\mu'} dz' \\ &\stackrel{1}{=} -\theta_{thr} e^{-S(t-t_f)/S\mu'_r} + \int_{z=0}^\infty f'\left(S(t-z)\right) \frac{1}{S\mu} e^{-St/S\mu} S\, dz \\ &= -\theta_{thr} e^{-(t-t_f)/\mu_r} + \int_{z=0}^\infty f(t-z)\, \frac{1}{\mu} e^{-t/\mu}\, dz \\ &= T(t; \mu, \mu_r) \end{aligned} \tag{45}$$

where step (1) sets $z' = Sz$, $dz' = Sdz$, $\mu' = S\mu$, and $\mu'_r = S\mu_r$. This concludes the proof that the LIF model, described as a set of linear filters, retains temporal scale covariance.

E Training setup

The model is implemented using the Norse library [11] and trained on a dataset generated with the Generated Event Response Data (GERD) framework [12]. The leaky integrator and leaky integrate-and-fire models are trained on NVIDIA A100 GPUs due to the high memory requirements, while the ReLU models are trained on NVIDIA V100 and 4090 cards. The models are trained using the Adam optimizer [13] with a learning rate of 5×10^{-4}. The batch sizes vary to account for the differing memory requirements. Each model is trained for 50 epochs and repeated 5 times.

All the initialization code and hyperparameters are available for reproduction at `https://jegp.github.io/nrf` and doi.org/10.5281/zenodo.16651826.

F Effects of batch normalization

To explore the hypothesis about the normalizing effect of the receptive field, a copy of the model presented in the main text was constructed with added batch normalization layers. Batch normalization normalizes signals with the following transformation [14]

$$\hat{x} = \frac{x_k - \mathbb{E}[x_k]}{\sqrt{\mathrm{Var}[x_k]}} \gamma + \beta \tag{46}$$

where $\mathbb{E}[x_k]$ and $\mathrm{Var}[x_k]$ are the mean and variance of the k-th feature, respectively, and γ and β are learnable parameters.

Layers following batch normalization are known to be less sensitive to distributional shifts both from the input data and the parameter changes [14].

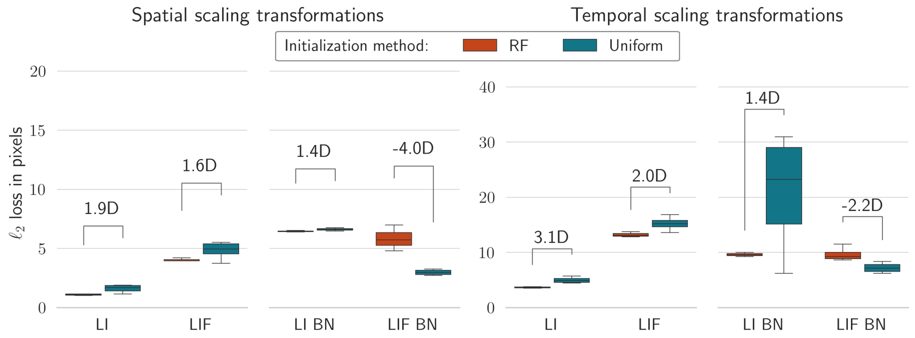

Supplementary Figure 4: Results of the spatial and temporal scaling experiments with and without batch normalization (BN). As in the main text, the model parameters are either initialized according to the spatio-temporal receptive fields or sampled uniformly.

Theoretically, this makes the model generalize better to unseen data and accelerate training at the cost of computational overhead. This overlaps partly with the theoretical properties of the scale-space representation, since they exhibit transformational covariance and improved generalization.

The addition of batch normalization could hypothetically cancel out some benefits of the scale-space initialization, as it would normalize the signals and provide part of the generalization properties. Figure 4 shows the comparison between models initialized with the spatio-temporal receptive fields and models initialized uniformly, this time with added batch normalization before each temporal activation layer in the blocks. Compared to the results in the main text, initialization with spatio-temporal receptive fields show significantly less improvement compared to the uniform initialization. In fact, for the spiking neuron models, it even hurts the performance. This is likely due to the fact that constant normalization reduces the sensitivity around the firing threshold, which risks silencing the neuron and quenching the gradients, thus leading to less effective training.

However, batch normalization is not compatible with neuromorphic architectures, since the batch normalization operation in (46) requires the synchronous computation of the mean and variance over the entire batch. As such, the comparison is a purely theoretical inquiry into the effects of the scale-space initialization.

Supplementary References

[1] Lindeberg, T. Covariance properties under natural image transformations for the generalized Gaussian derivative model for visual receptive fields. *Frontiers in Computational Neuroscience* **17**, 1189949 (2023).

[2] Lindeberg, T. A time-causal and time-recursive scale-covariant scale-space representation of temporal signals and past time. *Biological Cybernetics* **117**, 21–59 (2023). URL `https://link.springer.com/10.1007/s00422-022-00953-6`.

[3] Schoenberg, I. J. On variation-diminishing integral operators of the convolution type. *Proceedings of the National Academy of Sciences of the United States of America* **34**, 164–169 (1948).

[4] Lindeberg, T. *Scale-Space Theory in Computer Vision* (Springer US, Boston, M., 1994). URL `http://link.springer.com/10.1007/978-1-4757-6465-9`.

[5] Lindeberg, T. & Fagerström, D. *Scale-space with casual time direction*, vol. 1064 of *Lecture Notes in Computer Science*, 229–240 (Springer Berlin Heidelberg, Berlin, H., 1996). URL `http://link.springer.com/10.1007/BFb0015539`.

[6] Koenderink, J. Scale-time. *Biological Cybernetics* **58**, 159–162 (1988).

[7] Lindeberg, T. Time-causal and time-recursive spatio-temporal receptive fields. *Journal of Mathematical Imaging and Vision* **55**, 50–88 (2016).

[8] Lindeberg, T. A computational theory of visual receptive fields. *Biological Cybernetics* **107**, 589–635 (2013).

[9] Gerstner, W., Kistler, W. M., Naud, R. & Paninski, L. *Neuronal Dynamics: From Single Neurons to Networks and Models of Cognition* (Cambridge University Press, Cambridge, 2014). URL `http://ebooks.cambridge.org/ref/id/CBO9781107447615`.

[10] Eliasmith, E. & Anderson, C. H. Neural engineering: Computation, representation, and dynamics in neurobiological systems. *IEEE Transactions on Neural Networks* **15**, 528–529 (2004).

[11] Pehle, C.-G. & Pedersen, J. E. Norse - a deep learning library for spiking neural networks (2021). URL `https://zenodo.org/record/4422025`.

[12] Pedersen, J. E., Korakovounis, D. & Conradt, J. Gerd: Geometric event response data generation (2024). URL `http://arxiv.org/abs/2412.03259`. ArXiv:2412.03259 [cs].

[13] Kingma, D. P. & Ba, J. Adam: A method for stochastic optimization. *CoRR* **abs/1412.6980** (2014). URL `https://api.semanticscholar.org/CorpusID:6628106`.

[14] Ioffe, S. & Szegedy, C. Batch normalization: Accelerating deep network training by reducing internal covariate shift. In *Proceedings of the 32nd International Conference on Machine Learning*, 448–456 (PMLR, 2015). URL `https://proceedings.mlr.press/v37/ioffe15.html`.
